# Plasma Fatty Acid Composition Was Associated with Apelin Gene Expression in Human Adipose Tissues

**DOI:** 10.1155/2021/8846483

**Published:** 2021-10-06

**Authors:** Emad Yuzbashian, Golaleh Asghari, Nilofar Beheshti, Mehdi Hedayati, Maryam Zarkesh, Parvin Mirmiran, Afsoon Daneshafrooz, Alireza Khalaj

**Affiliations:** ^1^Nutrition and Endocrine Research Center, Research Institute for Endocrine Sciences, Shahid Beheshti University of Medical Sciences, Tehran, Iran; ^2^Department of Clinical Nutrition and Dietetics, Faculty of Nutrition Sciences and Food Technology, National Nutrition and Food Technology Research Institute, Shahid Beheshti University of Medical Sciences, Tehran, Iran; ^3^Cellular and Molecular Endocrine Research Center, Research Institute for Endocrine Sciences, Shahid Beheshti University of Medical Sciences, Tehran, Iran; ^4^Tehran Obesity Treatment Center, Department of Surgery, Shahed University, Tehran, Iran

## Abstract

**Background:**

Apelin is an adipokine with an intermediatory role in obesity and insulin resistance, which can be modified by dietary intake.

**Aims:**

In this study, we aimed to determine the association of the plasma fatty acid composition with apelin plasma concentration and gene expression in visceral (VAT) and subcutaneous (SAT) adipose tissues.

**Methods:**

In this cross-sectional study, we recruited 179 patients aged 19-75 years who were candidates for elective surgery. Through the surgery, SAT and VAT were collected to measure apelin gene expression. Anthropometric measurements, fasting blood samples, and dietary intakes were collected before surgery. Free fatty acids (FFAs) in fasting whole plasma were measured using gas chromatography with flame ionization detection. Linear regression models were used to estimate standardized *β* (STZ *β*) showing the association of individual and total FFAs with apelin gene expression after adjustment for potential confounding variables.

**Results:**

In multivariable analysis, we observed a significant positive association of total plasma free fatty acids (FFAs) (STZ *β* = 0.241, *P* = 0.006), saturated fatty acid (SFA) (STZ *β* = 0.336, *P* < 0.001), and monounsaturated fatty acid (MUFA) (STZ *β* = 0.313, *P* < 0.001) concentrations with apelin gene expression from VAT after controlling for age, sex, body mass index, homeostatic model assessment for insulin resistance (HOMA-IR), physical activity, and energy intake. In the SFA family, there was a direct association with plasma concentration of myristic acid (STZ *β* = 0.372, *P* < 0.001), pentadecanoic acid (STZ *β* = 0.252, *P* = 0.002), and heptadecanoic acid (STZ *β* = 0.407, *P* < 0.001) with apelin mRNA expression in VAT. There was no significant association between FFAs and apelin plasma concentration and SAT mRNA levels.

**Conclusions:**

In conclusion, circulating plasma FFAs, SFA, and MUFA had a positive association with apelin gene expression in VAT. It seems that plasma fatty acid composition may regulate apelin gene expression in VAT.

## 1. Introduction

Apelin is one of the adipokine family members, which is known as a highly conserved peptide and the endogenous ligand of APJ, a G-protein-coupled receptor [1, 2]. Apelin expressed and secreted from a variety of tissues, including mainly from adipose tissue as well as the central nervous system and the gastrointestinal tract, the heart, the lungs, and with endocrine, paracrine, and autocrine function [1]. Apelin is involved in a wide range of physiological pathways, including energy homeostasis, regulation of body fluid, and immune, gastrointestinal, and cardiovascular functions [3]. Current animal studies indicated that apelin had an insulin-sensitizing role and positively impacted glucose homeostasis [2]. The finding of a population-based study demonstrates that high apelin concentration was associated with reduced incidence of type 2 diabetes risk [4]; thus, it is implied that apelin may have a protective effect against metabolic disorders. Although some known physiological factors such as insulin and inflammation markers contribute to the regulation of apelin gene expression and plasma concentration, apelin regulation can be influenced by modifiable environmental factors [5, 6].

Free fatty acids (FFAs) have received remarkable interest in the context of metabolic disorders because they have potential roles in the inflammatory processes and gene expression of several organs [7, 8]. A mediatory effect of circulating FFAs is proposed in the glucose-stimulated insulin production/release, decreasing glucose tolerance and insulin secretion [4, 9]. Increased FFA concentration had an undesirable effect on insulin signaling [10]. Furthermore, observational and experimental studies indicated that the concentration of FFAs increases in obesity, which induces peripheral insulin resistance and decreased insulin secretion from pancreatic beta cells [11–14]. In addition to the impact of FFA concentration on glucose homeostasis, the evidence showed that adipokine concentration and gene expression could also be affected by the concentration of FFAs [15–17]. A direct association of erythrocyte membrane lauric and linoleic acids and an inverse association of total n-3, eicosapentaenoic (EPA), and docosahexaenoic acids (DHA) with adiponectin and leptin concentrations were reported [15]. Besides, evidence illustrates that EPA and DHA reduced the relative gene expression of leptin, whereas palmitic or oleic acid had no significant effect [18].

In this regard, to the best of our knowledge, there is no study to examine the association of apelin gene expression from adipose tissues and specific FFAs. Given the importance of regulation of apelin on glucose homeostasis and the capability of FFAs to change adipose tissue metabolism, we aimed to investigate the association of circulating individual FFAs with apelin circulation and gene expression from visceral and subcutaneous human adipose tissue.

## 2. Methods

### 2.1. Participants

This crosssectional study was conducted on a convenience sampling method of 176 adults aged 19–75 years who were admitted for elective surgeries to the Mostafa Khomeini and Khatam Al-Anbia hospitals, Tehran, Iran. We included participants if they were admitted to the hospital less than three days. We excluded those with a chronic illness affecting diet such as diabetes or cancer, using any lipid-lowering or antiobesity or antidiabetic medication, or women with pregnancy and lactation. Approximately 100 mg of subcutaneous adipose tissue (SAT) and visceral adipose tissue (VAT) samples were collected during their underlying surgeries in RNase- and DNase-free microtubes. Peripheral venous blood samples were also drawn after overnight fasting and were kept frozen at -80°C until analysis.

Ethics approval was obtained from the ethics committee of the Research Institute for Endocrine Sciences of the Shahid Beheshti University of Medical Sciences (NO: IR.SBMU.ENDOCRINE.REC.1396.483), and we conducted the current study in accordance with the Declaration of Helsinki and RIES institutional guidelines. Written informed consent was obtained from all participants.

### 2.2. Plasma Free Fatty Acids

The measurement of plasma FFAs was described in detail elsewhere [19]. Briefly, fatty acid methyl esters were added to the gas chromatograph (Varian 450, City, USA) with flame ionization detection to quantify individual fatty acids. The device was equipped with a cyanopropyl siloxane 88 (CP-Sill 88) coated with silicon-based polymers (polysiloxanes), polyethylene glycols, and solid adsorbent- (EU-) fused silica capillary column (100 m length, 0.25 mm internal diameter × 0.2 *μ*m film thickness). Nitrogen was used as the carrier gas. The temperature profile was optimized by achieving separated all peaks of interest. Peak retention times were identified by injecting known standards (37 components FAME mix, SUPELCO, USA) with known fatty acid composition. Then, a standard curve was prepared using the 3-point linear plot of different dilutions of the standard, and concentrations (mg/ml) of individual fatty acids of each sample were calculated.

### 2.3. Quantitative Real-Time Polymerase Chain Reaction of Apelin Expression

The process of real-time quantitative reverse transcription polymerase chain reaction (qRT-PCR) has previously been reported [5]. Total RNA was isolated from both adipose tissue samples according to manufacturer's protocol of TRIzol reagent (Invitrogen U.S.) and was treated with DNase I to remove traces of genomic DNA. We synthesized cDNA according to manufacturer's protocol by a commercial kit (BioFact, Korea). Real-time PCR (Rotor-Gene 6000 (Sydney, Australia)) was performed by SYBR Green Master Mix (Thermo Scientific, USA). For each gene, samples were run in duplicate for interassay control along with GAPDH and the nontemplate control (NTC). The expression of apelin in each sample was evaluated based on its threshold cycle (Ct), normalized to the Ct of the reference gene. The minimum information for publication of quantitative real-time PCR experiments (MIQE) were followed [20]. GAPDH was considered as the reference gene for normalization in the samples.

### 2.4. Covariate Measurements

We assessed habitual dietary intakes of participants using a valid and reliable semiquantitative food frequency questionnaire during an interview by a trained dietitian [21, 22]. We required participants to elaborate their frequency of consumption daily, weekly, or monthly through the past year. The United States Department of Agriculture food composition table was applied to estimate the nutrients and energy of each food item, except traditional Iranian foods, in which Iranian FCT was used.

Weight and height were measured, and body mass index (BMI) was calculated. Regular physical activity was estimated using the long forms of the reliable and validated Persian version of the International Physical Activity Questionnaire through interviews [23]. To measure energy expenditure, the concept of metabolic equivalents (MET) was used. The apelin plasma concentration was measured using the ELISA assay kit (ZellBio, Ulm, Germany), and inter- and intra-assay coefficient of variation (CV) was both 1.9%. Fasting plasma glucose (FPG) was measured using an enzymatic colorimetric method with glucose oxidase (Pars Azmoon Inc., Tehran, Iran). Inter- and intra-assay CVs were both 1.0% for FPG. Insulin was measured using an enzyme-linked immunosorbent assay (ELISA) with Mercodia AB kits (Uppsala, Sweden). Inter- and intra-assay CVs of insulin were 1.7% and 2.3%, respectively. The homeostatic model assessment of insulin resistance (HOMA-IR) was calculated according to the following formula: [fasting insulin (*μ*U/ml) × fasting glucose (mmol/l)]/22.5. Participants with HOMA − IR > 3.2 were considered to be insulin resistant.

### 2.5. Statistical Analysis

Characteristics of participants were reported by means (standard deviation; SD) and medians (25^th^–75^th^ percentile) for normally distributed and skewed continuous variables, respectively, or percentage for categorical variables. The distribution of the variables was checked by the histogram and Kolmogorov–Smirnov tests. Participants were divided based on their median total FFA concentration. Differences in biochemical and anthropometric between groups were tested by Student's *t*-test for normally distributed, Wilcoxon rank-sum test for skewed quantitative variables, and by chi-square test for categorical variables.

Multivariable linear regression models were applied to test the associations of total and individual fatty acids (continuous as the independent variable) with apelin gene expression and concentration (continuous as the dependent variable). The model was adjusted for age, sex, BMI, HOMA-IR, physical activity, and energy intake. Analyses were not stratified by sex or BMI because there were no meaningful interactions between total FFAs and these variables in the relationship with apelin gene expression and concentrations when the analysis was run with the inclusion of interaction terms in the regression models. Results from the linear regression models were reported as regression standardized *β* (STZ *β*), representing unitless variables to show the association of exposures with outcomes. Furthermore, we also reconsidered the goodness of fit and the normality distribution of residuals in the model. All statistical analyses were performed using the statistical package for the social sciences (SPSS) 15.0 software (SPSS Inc., Chicago, IL, USA), and *P* values < 0.05 were considered statistically significant.

## 3. Results

**Figure 1:**
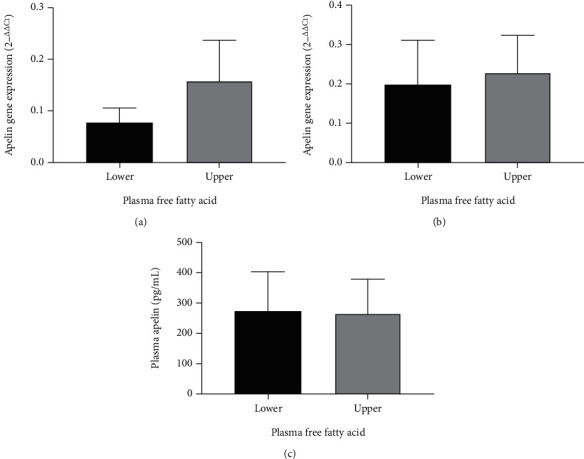
Distribution according to the upper and lower median of plasma free fatty acids regarding (a) apelin gene expression in visceral adipose tissue, (b) apelin gene expression in subcutaneous adipose tissue, and (c) apelin plasma concentration. Results are expressed as mean ± SD.

Participants presented a mean age of 41.0 years old, had a mean BMI of 35.6 kg/m^2^, and reported a mean total time physical activity energy expenditure of 1822 MET × min per week. The fasting plasma apelin concentration ranged from 117 to 561 ng/ml, with a mean of 266 ng/ml. A total of 176 participants were categorized into two groups: low FFAs (*n* = 88) and high FFAs (*n* = 88). There was no significant difference between upper and lower FFAs regarding apelin plasma concentration and gene expression in visceral and subcutaneous adipose tissue (Figure 1).

The biochemical and anthropometric characteristics of study participants are shown in Table 1. We compare two groups of low FFAs and high FFAs using *t*-test and Wilcoxon rank-sum test whenever these were appropriate. The values of BMI were higher in participants with higher FFAs compared to those in the lower FFAs (37.6 (9.9) vs. 33.7 (10.4) kg/m^2^; *P* = 0.011). The mean fasting plasma insulin concentration was significantly higher in the high FFAs than in the low FFAs participants (14.0 (10.1) vs. 10.6(11.2) *μ*U/ml; *P* = 0.034). As assessed using the HOMA-R formula, the prevalence of insulin resistance was significantly increased in participants with high FFAs compared with low FFAs ones (38.6 vs. 23.9%; *P* = 0.035).

The linear associations of plasma concentration of FFAs with apelin plasma concentrations and gene expression are presented in Table 2. In multivariable analysis, total plasma FFAs (STZ *β* = 0.241, *P* = 0.006) were directly associated with apelin gene expression from VAT. Furthermore, SFA (STZ *β* = 0.336, *P* < 0.001) and MUFA (STZ *β* = 0.313, *P* < 0.001) concentration also had a positive association with apelin mRNA levels from VAT after controlling for potential confounders. No significant associations of total FFAs and its subtypes with apelin concentration and SAT mRNA levels were observed.

The associations of plasma concentration of individual fatty acid with apelin plasma concentrations and gene expression are presented in Table 3. After adjusting for potential confounders, the plasma concentration of myristic acid (STZ *β* = 0.372, *P* < 0.001), pentadecanoic acid (STZ *β* = 0.252, *P* = 0.002), heptadecanoic acid (STZ *β* = 0.335, *P* < 0.001), cis-heptadecanoic acid (STZ *β* = 0.407, *P* < 0.001), and linolenic acid (STZ *β* = 0.156, *P* = 0.046) was positively associated with apelin mRNA levels in VAT, whereas plasma concertation of DHA was negatively associated with apelin gene expression in VAT. Plasma concentration of myristic acid (STZ *β* = 0.192, *P* = 0.013), pentadecanoic acid (STZ *β* = 0.186, *P* = 0.023), heptadecanoic acid (STZ *β* = 0.231, *P* = 0.003), and cis-heptadecanoic acid (STZ *β* = 0.265, *P* = 0.001) was positively associated with apelin mRNA levels in SAT. In addition, a significant negative association of eicosatetraenoic acid (STZ *β* = −0.164, *P* = 0.030) and a positive association of eicosapentaenoic acid (STZ *β* = 0.280, *P* < 0.001) with apelin plasma concentration were observed.

## 4. Discussion

In the present study of healthy adults, we found that total plasma FFA concentration was associated with the expression of an apelin in VAT after controlling for age, sex, BMI, HOMA-IR, physical activity, and energy intake. There was also a positive association of plasma SFA and MUFA with apelin gene expression from VAT. It should be noted that total FFAs and their subtypes were not associated with apelin concentration as well as SAT mRNA levels.

In recent years, apelin has extensively received attention concerning its role in the progression of insulin resistance. Apelin is expressed and released from adipose tissues, and its regulation is interrupted in obesity and insulin resistance [1]. The stimulatory role of apelin in glucose homeostasis in normal and obese insulin-resistant mice was also proposed [2]. Although there is no study to investigate the association of circulating FFAs with apelin in both serum/plasma concentration and gene expression level, human and animal experiments have demonstrated that apelin concentration and adipose gene expression changed in response to a high-fat diet [24]. Yang et al. illustrated that rats fed with a high-fat diet had higher apelin concentration as well as mRNA expression in adipose tissues [6]. In another study, Bertrand et al. showed that 10 weeks of high-fat diet mice lead to the upregulation of apelin concentration and gene expression in adipose tissue [25]. However, in the current study, we found that higher plasma FFAs were positively associated with apelin gene expression from VAT, unlike the null result observed for apelin concentration. This uncoupled companionship between apelin level in plasma and gene expression was reported in a study with feeding the high-fat diet in rats. After a 7-week intervention by a high-fat diet, the apelin mRNA level in SAT was higher than those in a standard diet, whereas apelin concentration did not change [26]. It seems that apelin gene expression in VAT is more prone to change in response to the manipulation of fat in a diet than apelin concentration. It is unclear whether adipose tissue contributes markedly to apelin plasma levels.

The results of animal studies support a relation between apelin gene expression and plasma SFAs and MUFAs. One study in rats showed that a diet with a high content of SFA increased apelin gene expression but not apelin plasma concentration [27]. Furthermore, Lasa et al. indicated that apelin concentration and gene expression in hamster feds with sunflower oil, as a source of MUFA, did not differ with standard-fed hamsters [28]. We also observed that myristic acid, pentadecanoic acid, and palmitic acid had a direct association with apelin gene expression in both VAT and SAT in the SFA family. Besides, plasma cis-heptadecanoic acid from the MUFA category had a positive association with apelin gene expression as well. The association of SFAs and MUFAs in the plasma with the transcription of apelin in the VAT suggests the contribution of fatty acid composition along with total FFA concentration in the regulation of apelin in fat depots.

Although studies showed a regulatory effect for PUFA families such as EPA, DHA, linoleic acid, and linolenic acid on the apelin plasma and gene expression in human [29] and animal studies [25, 27], we observed a null relationship. The lack of association between PUFAs and their subgroup plasma concentration may pertain to the difference of the various procedures that contributed to the absorption and metabolism PUFA intakes [30–32]. EPA has been reported to have an impact on the gene expression of apelin [33]. Furthermore, in high-fat-fed rats, EPA supplementation increased the apelin mRNA level in VAT [27]. Beyond the exploratory nature of the current study, which revealed association rather than causal linkage, findings warrant further examination using experimental and cohort studies to better understand the impact of plasma FFAs composition on adipokine expression.

Previous studies indicating that the fatty acid composition of plasma mirrors dietary fatty acid consumption and can accordingly be applied as an objective assessment of the type of fatty acids consumed by persons should be noted [34, 35]. However, since there are difficulties with calculating dietary fat consumption by dietary questionnaire with some inherent measurement error, the application of biomarkers is preferable [36]. For example, in addition to dietary intake, SFAs can be synthesized endogenously via acetyl CoA. Besides, as an abundant member of MUFAs, oleic acid can be produced endogenously through elongation and desaturation of SFAs.

Our study has some limitations. The crosssectional design of the study makes it unclear whether the higher fatty acids in plasma can increase apelin gene expression in adipose tissue. Another limitation of this study was the nonrandom selection of participants, and our sample was recruited from Tehran. Therefore, the results may not be representative of the population. The nature of the nonrandom increases the risk of selection bias and prevents generalization of the findings to the broader population. Despite adjustment for some potential confounders, several other confounders such as genetic background or race may still affect the association between fatty acid concentrations and apelin gene expression. This study also has its strength; it was the first study to provide data on human samples on the association of apelin gene expression and fatty acid concentrations.

## 5. Conclusion

All in all, the current study showed that total plasma FFA concentration was associated with increased VAT apelin gene expression. Furthermore, plasma concentrations SFA and MUFA were associated with higher apelin gene expression from VAT. The direct association of plasma FFA, SFA, and MUFA with apelin gene expression from VAT suggested that plasma fatty acid composition may play an indicatory role in regulating apelin gene expression in VAT.

## Figures and Tables

**Table 1:** The biochemical and anthropometric characteristics of study participants stratified by median of total plasma free fatty acids.

	Total	Total plasma free fatty acids		*P* value
		Lower median	Upper median	
Age (years)	41.1 (13.2)	42.9 (10.4)	39.2 (13.1)	0.069
Female (%)	75.0	79.5	71.5	0.164
Body mass index (kg/m^2^)	35.6 (10.3)	33.7 (10.4)	37.6 (9.9)	0.011
Fasting plasma glucose (mg/d)	88.8 (18.7)	88.2 (19.2)	89.4 (18.3)	0.673
Fasting plasma insulin (*μ*U/ml)	12.3 (10.7)	10.6 (11.2)	14.0 (10.1)	0.034
Insulin resistant (%)	31.3	23.9	38.6	0.035
Physical activity (MET/week)	567 (163-1531)	540 (179-1793)	585 (107-1415)	0.714
*Dietary intake*				
Total energy intake (kcal)	2881 (1000)	2812 (939)	2951 (1059)	0.356
Fat (% energy)	31.0 (6.0)	31.2 (6.0)	30.9 (6.1)	0.727
Saturated fatty acids (% energy)	9.6 (2.6)	10.0 (2.7)	9.3 (2.6)	0.116
Monounsaturated fatty acid (% energy)	10.2 (2.2)	10.4 (2.2)	10.1 (2.3)	0.343
Polyunsaturated fatty acid (% energy)	6.2 (1.7)	6.4 (1.6)	6.2 (1.7)	0.437
*Plasma fatty acids*				
*Saturated fatty acids*	2.2 (1.4)	1.32 (0.54)	3.12 (1.55)	<0.001
Lauric acid, C:12	0.07 (0.07)	0.06 (0.06)	0.09 (0.07)	<0.001
Myristic acid, C:14	0.32 (0.35)	0.20 (0.14)	0.45 (0.45)	<0.001
Pentadecanoic acid, C15	0.10 (0.13)	0.04 (0.07)	0.16 (0.15)	<0.001
Palmitic acid, C:16	0.86 (0.51)	0.56 (0.24)	1.17 (0.54)	<0.001
Heptadecanoic acid, C:17	0.28 (0.51)	0.08 (0.13)	0.46 (0.66)	<0.001
Stearic acid, C:18	0.50 (0.31)	0.33 (0.12)	0.68 (0.33)	<0.001
Lignoceric acid, C:24	0.07 (0.09)	0.04 (0.07)	0.10 (0.09)	<0.001
*Monounsaturated fatty acid*	1.9 (1.9)	0.88 (0.42)	2.92 (2.27)	<0.001
Palmitoleic acid, C16:1	0.29 (0.19)	0.21 (0.12)	0.37 (0.21)	<0.001
Oleic acid, C18:1	1.42 (1.77)	0.36 (0.38)	2.21 (2.20)	<0.001
Cis heptadecanoic acid, C17:1	0.19 (0.57)	0.05 (0.15)	0.34 (0.77)	<0.001
*Polyunsaturated fatty acid*	4.7 (3.90)	2.62 (1.02)	6.79 (4.52)	<0.001
Linoleic acid, C18:2	3.40 (3.66)	1.60 (0.87)	5.10 (4.40)	<0.001
Linolenic acid, C18:3	0.15 (0.09)	0.12 (0.08)	0.18 (0.09)	<0.001
Gamma linolenic acid, C18:3	0.13 (0.09)	0.10 (0.08)	0.16 (0.08)	<0.001
Eicosadienoic acid, C20:2	0.02 (0.04)	0.01 (0.03)	0.03 (0.05)	0.022
Eicosatetrienoic acid, C20:3	0.27 (0.15)	0.23 (0.11)	0.32 (0.16)	<0.001
Arachidonic acid, C20:4	0.45 (0.29)	0.35 (0.21)	0.10 (0.09)	<0.001
Eicosapentaenoic acid, C20:5	0.07 (0.09)	0.04 (0.07)	0.10 (0.09)	<0.001
Docosahexaenoic acid, C22:6	0.20 (0.12)	0.16 (0.09)	0.24 (0.13)	<0.001

Data are presented as mean (SD) or median (25-75 percentile) for quantitative variables according to their distribution and percent for categorical variables.

**Table 2:** Association of plasma fatty acids with apelin plasma concentration and adipose tissues gene expression.

	Visceral		Subcutaneous		Plasma	
	STZ *β*	*P* value	STZ *β*	*P* value	STZ *β*	*P* value
Total plasma FFA	0.241	0.006	0.040	0.655	-0.074	0.389
Saturated fatty acids	0.336	<0.001	0.160	0.068	-0.058	0.496
Monounsaturated fatty acid	0.313	<0.001	0.130	0.148	-0.099	0.249
Polyunsaturated fatty acid	0.116	0.194	-0.064	0.475	-0.052	0.545

The model was adjusted for age, sex, body mass index, HOMA-IR, physical activity, and energy intake. Multivariable linear regression analyses was performed, and standardized (STZ) *β* were reported.

**Table 3:** The associations of plasma concentration of individual fatty acid with apelin plasma concentrations and adipose tissues gene expression.

	Visceral		Subcutaneous		Plasma	
	STZ *β*	*P* value	STZ *β*	*P* value	STZ *β*	*P* value
Saturated fatty acids						
Lauric acid, C:12	0.068	0.383	-0.070	0.370	0.066	0.361
Myristic acid, C:14	0.372	<0.001	0.192	0.013	-0.023	0.751
Pentadecanoic acid, C15	0.252	0.002	0.186	0.023	0.052	0.499
Palmitic acid, C:16	0.035	0.659	-0.050	0.523	-0.055	0.448
Heptadecanoic acid, C:17	0.335	<0.001	0.231	0.003	-0.011	0.878
Stearic acid, C:18	-0.017	0.832	-0.094	0.232	-0.125	0.085
Lignoceric acid, C:24	-0.028	0.724	0.035	0.652	0.032	0.660
MUFA						
Palmitoleic acid, C16:1	0.038	0.634	0.055	0.484	-0.067	0.362
Oleic acid, C18:1	0.079	0.316	-0.002	0.982	-0.077	0.285
Cis heptadecanoic acid, C17:1	0.407	<0.001	0.265	0.001	-0.042	0.565
PUFA						
Linoleic acid, C18:2	0.042	0.588	-0.080	0.300	-0.044	0.543
Linolenic acid, C18:3	0.156	0.046	0.077	0.322	0.008	0.910
Gamma linolenic acid, C18:3	0.095	0.252	-0.090	0.268	-0.137	0.070
Eicosadienoic acid, C20:2	-0.025	0.747	-0.057	0.463	-0.139	0.051
Eicosatetrienoic acid, C20:3	0.028	0.736	-0.072	0.382	-0.164	0.030
Arachidonic acid, C20:4	-0.131	0.114	-0.095	0.248	0.057	0.455
Eicosapentaenoic acid, C20:5	-0.049	0.551	0.004	0.965	0.280	<0.001
Docosahexaenoic acid, C22:6	-0.156	0.048	-0.064	0.412	-0.054	0.460

The model was adjusted for age, sex, body mass index, HOMA-IR, physical activity, and energy intake. Multivariable linear regression analyses was performed, and standardized (STZ) *β* were reported.

## Data Availability

The datasets analyzed during the current study are available from the corresponding author on reasonable request.

## Abbreviations

VAT: Visceral adipose tissues
SAT: Subcutaneous adipose tissues
BMI: Body mass index
CV: Coefficients of variation
ELISA: Enzyme-linked immunosorbent assay
FPG: Fasting plasma glucose
MET: Metabolic equivalents task
SD: Standard deviation
SPSS: Statistical Package for the Social Sciences program
STZ *β*: Standardized *β*
FFAs: Free fatty acids
SFA: Saturated fatty acid
MUFA: Monounsaturated fatty acid
PUFA: Polyunsaturated fatty acid.

## Additional Points

*Highlight*. A significant positive association of total plasma and circulating plasma FFAs, SFA, and MUFA with apelin gene expression in VAT was observed. There was no association between plasma FA and circulating apelin. It seems that plasma fatty acid composition may regulate apelin gene expression in VAT.
